# Breakfast Clubs: Starting the Day in a Positive Way

**DOI:** 10.3389/fpubh.2015.00172

**Published:** 2015-07-08

**Authors:** Pamela Louise Graham, Riccardo Russo, Margaret Anne Defeyter

**Affiliations:** ^1^Department of Psychology, Northumbria University, Newcastle upon Tyne, UK; ^2^Department of Psychology, University of Essex, Colchester, UK

**Keywords:** children, breakfast, behavior, school, physical activity

## Abstract

Breakfast clubs are widely promoted as having a beneficial impact on children’s behavior at the start of the school day, which can be conducive to their learning within the classroom. However, the few available studies that have considered the impact of breakfast club attendance on children’s behavior have yielded mixed results and no studies to date have directly observed children’s behavior within the breakfast club setting. Using a combination of real-time observation and filmed breakfast club footage, the aims of the current study were to: (1) devise a set of observational criteria appropriate for use in the breakfast club setting; (2) investigate the occurrence of both positive and negative behaviors. A sample of 30 children aged between 3 and 11 years were recruited from 3, opportunistically sampled primary school breakfast clubs in the North East of England, UK. The behaviors they displayed within the breakfast club setting on two separate days were observed and coded for subsequent analysis. Results of the investigation showed that children’s behavior could be classified into three positive and three negative behavioral categories. Using these categories to code children’s behavior as they engaged in breakfast club showed that children displayed more positive than negative behaviors within the breakfast club setting and this was the case regardless of the type of activity (i.e., quiet or boisterous) children were involved in. Findings are discussed in relation to breakfast club policy, implementation, and evaluation.

## Introduction

The amount of time a child spends concentrating on a task is probably the most important aspect of learning (1); thus, it is plausible to argue that persistent disruptive behavior has the potential to be detrimental to children’s long-term academic performance as it reduces the amount of time that children spend focusing on school-related tasks (2). Moreover, children’s behavior in the classroom is likely to be particularly important at the start of the school day as this is the time that schools in the UK tend to teach core subjects such as English and Mathematics (3).

Prior to the start of formal learning in the classroom, some children across the UK choose to spend time in a school breakfast club. School breakfast clubs are a type of before school provision that typically take place on the school premises immediately prior to the start of the formal school day. While there are a variety of breakfast club models in operation, in general, breakfast clubs provide children with the opportunity to consume a nutritious breakfast in an adult-supervised environment while in the company of their peers. Additionally, many clubs also offer children opportunities to partake in structured activities before they move on to class for the start of the formal school day [for a review, see Defeyter et al. (4)]. There is a common perception among some educators and advocates of school breakfast provision that attendance at school breakfast clubs can have a beneficial impact on children’s behavior at the start of the school day; with the perception that breakfast clubs provide a calm period for children prior to the formal school day (5–7). However, very few studies have directly investigated the potential association between breakfast club attendance and children’s behavior. The few studies that are available have yielded mixed results and have tended to focus on children’s behavior after breakfast club rather than within the breakfast club setting.

In an early study of an in-class breakfast program in the USA (8), the on- and off-task behaviors of a group of male adolescents were recorded while they participated in a vocational welding class. To be classed as on-task, adolescents had to be either collecting or setting up welding equipment, welding within a designated booth, or returning equipment to the correct place. Behavior was observed across 40 school days and adolescents received “a nutritious breakfast” (p. 4) as they entered the classroom on 20 of these days. Results of the investigation showed that on-task behavior increased when adolescents consumed a school breakfast.

On the contrary, a large-scale UK investigation into school breakfast clubs identified a negative relationship between school breakfast club attendance and the behavior of children and adolescents (9). Data were collected using the Strengths and Difficulties Questionnaire (10), which measures aspects of emotional, hyperactive/inattentive, and social behaviors. Adolescents, aged 11–16 years, provided self-report ratings of their own behaviors while class teachers rated the behaviors of primary school children aged 4–11 years. Analysis showed that children and adolescents who had attended a breakfast club were more likely to have borderline or abnormal behavioral scores compared to those who had not attended breakfast club. Further, anecdotal evidence collected from teachers and observations made by the research team suggested that some children were more difficult to settle following breakfast club attendance and this may have been a result of inadequate supervision and an allowance of rough and boisterous play within the breakfast club setting.

More recently, the impact of breakfast club attendance on behavior was considered as part of a randomized controlled trial carried out on a sample of year 5 and year 6 children (aged 9–11 years) from 111 primary schools in Wales, UK (11). At baseline and 12-months post implementation of breakfast clubs, teachers completed the Strengths and Difficulties Questionnaire (10) for a subsample of 10 randomly selected children from each participating school. The hyperactivity/inattention subscale was of particular interest to the authors as they deemed this to be related to children’s breakfast consumption and on-task behavior based on previous research. Analysis showed no significant difference between children in the control and intervention conditions in terms of teacher-reported hyperactivity/inattention, suggesting that, in this instance, breakfast club attendance did not appear to influence children’s classroom behavior.

Overall, there is currently no consensus on the impact of breakfast club attendance on children’s behavior. Moreover, no published research exists to describe children’s behavior within the breakfast club setting; research to date has focused predominantly on children’s ability to remain on task in the classroom following breakfast club attendance or reports on their behavior in general according to scores calculated from the Strengths and Difficulties Questionnaire (10). It has been suggested that “observational data have greater external or ecological validity than behavior rating scales, as they provide a measurement of the behavior as it is actually occurring in the school context” (12) (p. 360). However, as there are no published data on children’s behavior within breakfast club, there are currently no available observational criteria that would allow children’s behavior in breakfast club to be rated. The current paper presents two studies to address the lack of research surrounding breakfast clubs and children’s behavior. The aim of Study 1 was to devise a set of observational criteria that would be relevant to children’s behavior within the breakfast club setting. These criteria were then used in Study 2 to investigate the display of positive and negative behaviors by children as they participated in breakfast club activities.

## Study 1

### Method

#### Participants

Following approval from the University Ethics Committee, children from two primary school breakfast clubs based in the North East of England were invited to participate in the current study. Both participating breakfast clubs were opportunistically sampled. The breakfast clubs were open to children aged 5–11 years from 8:00 a.m. until 8:50 a.m., Monday to Friday, during school-term time only and both were of similar size accommodating around 10 children per day. Children at both schools were offered a variety of breakfast items including cereal, toast, fruit, and juice. School 1 also offered children hot items, such as beans and eggs and School 2 offered children pancakes. Additionally, breakfast clubs provided children with a range of activities to participate in. School 1 offered children opportunities to engage in pretend play and table top activities, such as jigsaws and drawing. School 2 also offered table top activities and toys as well as physical activities, such as skipping and ball games. Attendance at both clubs incurred a charge of £2.50 per child per day.

It was not possible to obtain demographic information on individual children who participated in the current study, but participating schools were similar across a number of characteristics. Both schools resided in the same town in the North East of England, UK, in areas that were made up of a high percentage of White British residents (School 1 and School 2 local areas: 97%; national average: 79.8%). Both schools were in rather deprived areas as indicated by a high percentage of working age people claiming key state benefits (School 1 local area: 22%; School 2 local area: 25%; national average: 15%) and an above average percentage of children claiming free school meals (School 1: 21%; School 2: 39.9%; national average:18%).

Use of an opt-in method of parental consent resulted in only a small number of children returning their forms to school giving consent for them to participate in the study, so it was not possible to observe the behavior of all children within the breakfast clubs. The sample of children observed consisted of nine females and three males, aged 6:11–10:4 (mean age = 8:4). Five children were from School 1 and seven children were from School 2. All children attended breakfast club on at least two school days per week during the 2-week study period and were reported by staff to have attended breakfast club for most weeks during the school year.

#### Equipment

A small MP3 player with in-ear headphones was used to allow the observer to discretely listen to a pre-recorded track that played an audible beep at 20 s intervals. The sound was used to alert the researcher to the end of each observation and recording interval.

#### Procedure

Opt-in consent was obtained from school head teachers, breakfast club staff, and parents/carers of children attending breakfast club. Prior to observations taking place, the observer briefed children on the nature of the research, obtained verbal assent from each child, and explained their rights to confidentiality and to withdraw from the study at any time.

In an effort to reduce participant reactivity, one observer attended breakfast club for five consecutive school days prior to the commencement of observations. The observer arrived before all children and consistently sat in the same area of the breakfast club each day where all breakfast club activities could be viewed without imposing on children’s breakfast consumption or activity involvement.

On observation days, each target child was observed across a 45-min period with up to five children being observed on 1 day. Each child was observed for 20 s, then the observer spent 20 s noting down the dominant behavior exhibited by the target child during the 20-s observation interval. At the end of the recording interval, the observer moved on to observe the next target child and continued to observe each child in the same manner for the duration of the breakfast club session. On occasions when fewer than five children were observed, the observer ensured that timings remained consistent, so there was always the same break of 2 min and 40 s between each observation/recording interval allocated to each child. When all observations were complete, children were verbally debriefed and were given a written debrief form to take home to inform their parent or carer that the research had taken place.

### Results

Initially, all behaviors included on the observation records were categorized as either positive or negative. Positive behaviors were classified as those behaviors deemed appropriate within the context of the breakfast club; children’s engagement in these behaviors did not result in another person being hurt or upset and did not lead to children being reprimanded by staff. Conversely, behaviors coded as negative were reprimanded by staff and/or resulted in conflict between peers. Inattentive behaviors, such as wandering the room, were also considered negative as previous research has linked such behavior to delinquency (13).

Closer consideration of the behaviors included in the positive and negative categories showed that further distinctions could be made between behaviors within these categories. For the positive category, three subcategories of behavior were apparent: (1) *On-task independent*: a child quietly engaged in an activity and not involved in any kind of social exchange. A child standing back and watching the behavior of another in the room was also coded under this category as watching others is a way of gathering information and therefore this could be considered exploratory behavior (14); (2) *On-task social*: a child engaged in an activity while chatting to another person; (3) *Off-task social*: a child engaged in conversation with another person while not carrying out any other activity. Similarly for the negative category, three distinct subcategories of behavior emerged: (1) *Off-task antisocial*: victimizing or arguing with another person; (2) *Active distraction*: disruptive or boisterous behavior, such as shouting, inappropriately or running around in a space where running is not permitted; (3) *Passive distraction*: lack of focus on anything in particular. A *Not Visible* category was also included aside from the positive and negative behavior categories for when children left the room and their behavior was unobservable. Details of the behaviors listed under each category are presented in Table 1.

**Table 1:** **Behavioral categories**.

	Behavioral categories	Example behaviors
Positive behaviors	On-task independent	Pouring juice
	On-task social	Playing catch with peer
	Off-task social	Chatting to peers
Negative behaviors	Off-task anti-social	Conflict over game
	Active distraction	Crawling around the floor
	Passive distraction	Wandering around the room

### Brief discussion

The aim of Study 1 was to develop a set of observational criteria that would be appropriate for observing children’s behavior within breakfast club settings. The behaviors exhibited by 12 children within two breakfast clubs were coded into 6 behavioral categories; 3 positive and 3 negative. However, the criteria were devised based on the behavior of a small sample of children from two primary school breakfast clubs; therefore, further investigation through observations of children’s behavior in different breakfast clubs is warranted to determine whether the observational criteria are relevant to breakfast clubs beyond those observed in the current study. Moreover, although a multitude of behaviors were observed using the time sampling method where behaviors were recorded at 20 s intervals, on many occasions, the same behaviors were recorded across two intervals suggesting that a longer observation time could be used. Children’s behavior during play does not tend to change frequently; therefore, a 5-min observation interval is acceptable for observing children’s behavior in a play-based situation (15). There are currently no guidelines regarding an optimal observation period for children’s behavior in breakfast clubs but as children spend time playing in breakfast clubs, use of 5-min observation intervals in future investigations should be considered.

A further methodological consideration that should be made in future observational studies of breakfast clubs concerns the way in which children’s behaviors are observed. In a recent review of the literature surrounding breakfast and children’s behavior (16), it was argued that real-time observations, where behaviors are monitored and recorded as they occur, can be challenging and prone to observer fatigue. It was further recommended that video recordings should be employed where possible as the ability to pause and replay footage increases the accuracy of observations and allows behavior to be coded by multiple observers therefore improving reliability of reported findings.

Finally, the opt-in method of consent used in the present study resulted in a small sample size as a number of children did not return parental consent forms. “Failure to return forms is typically due to parents’ busy lives rather than disapproval of their child’s participation” (17) (p. 72); thus, there is a possibility that this was the case in the current study as the forms distributed to parents and carers asked them to return the forms to specify whether or not they wanted their child to be included in the study and no parents returned forms opting their child out suggesting that they did not disagree with their child’s participation in the research but did not get around to returning the form to opt them in either. In future, it may be useful to adopt an opt-out method of consent in an effort to increase the number of children involved in the research and to reduce the burden placed on parents to return forms to school.

The behavioral criteria devised in Study 1 were used to observe children’s behavior in Study 2 to determine whether there was a difference between the number of positive and negative behaviors exhibited by children within breakfast club and to consider whether the activities that children engaged in during breakfast club made a difference to the behaviors they displayed. Taking into consideration the methodological issues that were raised within Study 1, Study 2 adopted an opt-out method of consent, and children’s behaviors were filmed within the breakfast club setting so that subsequent coding could be completed.

## Study 2

### Method

#### Participants

Following ethical approval from the University Ethics Committee, the head teacher of a primary school based in the North East of England provided consent for children’s behavior to be filmed in the school’s breakfast club. It was not possible to obtain demographic information on individual children who participated in Study 2 of the current investigation but the characteristics of the school, which will be referred to as School 3, and the local area that the school resided in are subsequently outlined. School 3 was based in a predominantly White British area (98%) of the North East of England. The local area had a high percentage of working age people claiming key state benefits (School 3 local area: 19%; national average: 15%) and an average percentage of children claiming free school meals (School 3: 18.2%; national average: 18%).

The breakfast club in School 3 was available to children aged 3–11 years from 7:30 a.m. until 8:50 a.m., Monday to Friday, during school-term time only. For children who participated in breakfast club from 7:30 a.m., attendance incurred a cost of £2.50 per child per day whereas attendance from 8:00 a.m. incurred a cost of £2:00 per child per day. Children were offered a variety of breakfast items including cereal, toast, juice, and fruit. They were also able to spend time engaging in table top activities, such as drawing and construction, and physical activities, such as skipping and ball games.

The head teacher acted *in loco* parentis to allow all children attending breakfast club to participate in the research unless they were opted out by their parents. Subsequently, no parents opted their children out of the study and all children were happy to participate. Breakfast club staff also provided written consent to be filmed. Twenty-five children aged between 3 and 11 years were filmed within the breakfast setting during the seven-day study period. The behavior of all children in attendance was filmed but it was necessary to exclude seven children from the coding element of the study. Two children were excluded as they arrived at breakfast club in time to participate in only one activity, so it was not possible to look at their behavior across different activities. A further five children were withdrawn as they only attended breakfast club on one occasion during the study period. The behavior of the remaining 18 children (9 males and 9 females) was observed and coded according to the procedure outlined below. It was not possible to calculate children’s exact ages as the adoption of an opt-out method of consent meant that children’s dates of birth could not be obtained.

#### Equipment

Two VIO POV-HD cameras (Extreme Technologies, Minneapolis, MI, USA) were set up within each of the two rooms where breakfast club sessions took place. Each camera was disguised within a box file attached to a lever-arch file, so only the camera lens was visible through an existing hole in the lever-arch file.

#### Procedure

Approximately 10 min before the start of breakfast club, two cameras were set up in the Breakfast Club Room where children spent up to 45 min participating in desk top activities, such as board games and Lego. The school’s breakfast club began in this room at around 7:30 a.m. each day and children remained there for around 45 min before moving into the school hall, where they spent around 45 min having breakfast and participating in physical activities, such as ball games. The cameras were positioned in each room so that children’s behavior could be filmed without the presence of the cameras being obvious to children and so that all areas were in camera shot. Filming began in each room before children entered and was terminated after children had left so the researcher would not be seen by the children. Filming was conducted according to the outlined procedure on seven school days. When filming was completed all children were verbally debriefed and were given a sticker and a pencil as a token of appreciation for their participation in the research. Children were also given a written debrief letter to take home to inform their parent or carer that the study had been completed.

#### Behavioral Coding

The recordings of children’s behavior within the breakfast club sessions were subsequently coded according to the three positive (On-task Independent; On-task Social; Off-task Social) and three negative (Off-task Anti-social; Active Distraction; Passive Distraction) behavioral criteria devised in Study 1. As children arrived at breakfast club at different time points, the behavior of each child was observed and recorded individually. For each child, the time that they arrived at breakfast club was noted then that child’s behavior was observed and the footage was paused at 5 min intervals. The behavior that children were engaged in at the 5 min interval point was recorded along with any additional relevant notes such as unexpected incidents observed that might have influenced children’s behavior. The behavior of each child was observed across the entire duration of one breakfast club session.

#### Second Coding

In order to determine the accuracy of the first observer’s interpretation of children’s behavior, just over 10% of the data (i.e., 2/18 participants) were coded by a second observer. Analysis showed that there was moderate agreement between the first and second observer (Kappa coefficient = 0.59; *p* < 0.001).

The main discrepancy between the two observers’ interpretations of children’s behavior was concerned with whether a child engaged in a task while not appearing to speak to anyone could be considered to be working independently if they were seated at a table with other children who were obviously interacting with peers at the same table, i.e., the observed child could have been listening to the conversation without actively engaging in it. This difficulty in interpretation emerged as a result of the cameras used to record children’s behavior not being able to detect sound in detail enough to hear children’s interactions; thus, observations were based on children’s motor behaviors. For this reason, children’s behaviors were analyzed based on positive and negative behavioral groupings as these were easier to interpret than more detailed behavior (i.e., independent vs. social behaviors).

#### Analysis

Wilcoxon-signed rank tests were used to compare the mean numbers of positive and negative behaviors within the Breakfast Club Room and the Hall. Wilcoxon-signed rank tests were also used to determine whether there was a difference between rooms in terms of the number of positive behaviors displayed by children and the number of negative behaviors displayed in each room. Further analysis was carried out to consider whether children’s behavior changed across the duration of the breakfast club session. The first, middle, and last intervals recorded for each child across the duration of the entire breakfast club session were scored with 1 given for a positive behavior and 0 given for a negative behavior. Scores were then compared using Wilcoxon-signed rank tests to identify any differences between the first and middle interval, the middle and last interval, and the first and last interval.

### Results

Analysis of children’s behavior within the breakfast club room showed that children displayed significantly more positive (*Mdn* = 1) than negative (*Mdn* = 0) behaviors while engaged in quiet activities (*Z* = 3.89; *p* < 0.001). The same pattern of results emerged for the Hall with children displaying significantly more positive (*Mdn* = 1) than negative (*Mdn* = 0) behaviors when engaged in more boisterous activities (*Z* = 3.72; *p* < 0.001).

Further, comparisons made between the Breakfast Club Room and the Hall showed that there was no significant difference between the number of positive behaviors (*Z* = 1.49; *p* = 0.13) or the number of negative behaviors (*Z* = 1.49; *p* = 0.13) displayed in each room.

Finally, when children’s behaviors were compared across the duration of the breakfast club sessions, there were no significant differences between the first and middle interval (*Z* = 0.27; *p* = 0.78), the middle and last interval (*Z* = 1.22; *p* = 0.22), and the first and last interval (*Z* = 1.34; *p* = 0.18).

### Brief discussion

The aim of Study 2 was to investigate the occurrence of positive and negative behaviors within the breakfast club setting and to determine whether the type of activity that children engaged in (i.e., quiet or active) made any difference to the number of positive and negative behaviors displayed. Results showed that children displayed more positive than negative behaviors while engaged in quiet and active tasks, and there were no significant differences between activities in terms of the number of positive or negative behaviors displayed, i.e., children did not display more positive or negative behaviors in one type of activity compared to the other. Moreover, children’s behavior did not appear to improve or deteriorate across the duration of the breakfast club session (as indicated by comparisons made between behavior displayed during the first, middle and last interval of breakfast club). While the current findings lend support to ideas put forward by researchers, practitioners, and policymakers (5–7) to suggest that breakfast clubs can provide children with a positive start to the day, they do not support previous research suggesting that breakfast club attendance can be detrimental to children’s behavior (9). However, it is important to acknowledge that the level of supervision within breakfast clubs might be a crucial factor in determining children’s behavior as it has previously been argued that poor behavior in breakfast clubs might be a result of inadequate supervision (9). The breakfast club observed in the current study was supervised by two members of school staff who were responsible for between 11 and 16 children during each breakfast club session. Furthermore, each breakfast club session recorded followed the same routine each day with children always being offered quiet, table top activities in the Breakfast Club Room followed by breakfast and active tasks in the Hall. Any displays of inappropriate behavior were dealt with quickly by breakfast club staff and even when more boisterous games, such as dodge ball, were played, the breakfast club staff ensured that the noise level was kept under control. It has been proposed that routines are key to the promotion of positive behavior as they ensure a predictable environment and encourage rule-governed behavior (18). It might be the case that the combination of adequate staffing and a fixed routine in the breakfast club observed in the current study led to the predominant display of positive behaviors suggesting that routine and adequate staffing are key elements that require consideration in breakfast club delivery.

Unfortunately, it was not possible in the present study to investigate whether the positive behaviors displayed by children in breakfast club continued into the classroom. Ethical approval was provided by the University Ethics Committee to allow filming to take place in classrooms following breakfast club but it was stipulated that *all* children must provide consent and must obtain parental consent before filming in class can take place. However, four parents of children who did not attend breakfast club opted their children out of the study and seven children did not consent to their behavior being filmed within the class, so it was not possible to conduct this aspect of the study as planned. Filming behavior is said to provide a “more accurate and ecologically valid” (16) (p. 23) measure of behavior than real-time observations. However, the current study demonstrates that the adoption of this method in schools is challenging.

## Conclusion

The current study showed that children’s overall behavior within the breakfast club setting is consistently positive with few negative behaviors observed. Moreover, it was evident in the current study that when routines are structured and breakfast club staff are consistent in their expectations of behavior children can be allowed to engage in quiet or active tasks with no significant detriment to their behavior. This has important implications for policymakers and practitioners looking for ways to encourage children to engage in physical activity. The current findings suggest that breakfast clubs could offer a structured environment in which children can be active for at least 30 min before the start of the school day. This would contribute substantially to helping children achieve the minimum 60 min of physical activity recommended by the Chief Medical Officer (19). Further research is needed to ascertain whether children’s positive behavior in breakfast club carries over into the classroom and whether the type of task that children engage in within breakfast club has an effect on their subsequent ability to pay attention to their work in class.

Finally, the current study proposes a set of criteria suitable for the observation of behavior within breakfast clubs. While the criteria were only used within three schools in the present investigation, children were observed engaging in a multitude of activities within the breakfast club settings of different schools and the criteria suitably encompassed all behaviors that were displayed. The use of a second coder in Study 2 highlighted that an inability to detect children’s voices through filmed observation footage makes fine coding of children’s behavior difficult. However, the proposed observational criteria were successful in allowing observers to distinguish and agree upon displays of positive and negative behaviors. Further investigations are needed to validate the observational criteria and to test their suitability for use across different breakfast club models, for example, when breakfast is served within the classroom or within a community setting.

## Conflict of Interest Statement

This research was sponsored by Kellogg’s.
